# Supplementary material for: Avoiding the bullies: The resilience of cooperation among unequals
Source: PLoS Comput Biol. 2021 Apr 7;17(4):e1008847. doi: 10.1371/journal.pcbi.1008847 (PMC8055019; doi:10.1371/journal.pcbi.1008847)
Supplement: S1 Text — Supporting information containing additional description of game notation, review of related work (Table A), and mathematical analyses of the static game, the dominant solvable breakpoint, and the breakdown of conventions. It also contains additional simulation results and additional figures for evolution of strategies (Fig A), network evolution (Fig B), partner choice and dynamic ranks (Fig C), comparison of timescales (Fig D and Fig E), cycle length (Fig F), total payoffs (Fig G), inequality (Fig H), payoffs by rank (Fig I), and cycle length for different population sizes (Fig J). (PDF) [file pcbi.1008847.s001.pdf]

S1 Text – Supplementary Information

Avoiding the bullies: The resilience of cooperation among unequals

by Michael Foley, Rory Smead, Patrick Forber, Christoph Riedl

PLOS Computational Biology

Note on games and representation

The baseline representation for the strategic interactions we aim to investigate is a game of conflict. By game of conflict we mean the generic set of symmetric 2×2 games where payoffs are strictly ordered such that there are two strict asymmetric Nash and one symmetric mixed Nash equilibria [*1*]. Given the payoff matrix below (eq 7), that payoff ordering condition is $hd > dd > dh > hh$. Traditional models that use infinite populations with random-mixing have a single stable point: the mixed Nash equilibrium. In the literature most descriptions of hawk-dove, snowdrift, chicken, and anti-coordination games count as generic games of conflict.

$$\begin{array}{c c} & \begin{array}{cc} \text{hawk}_j & \text{dove}_j \end{array} \\ \begin{array}{c} \text{hawk}_i \\ \text{dove}_i \end{array} & \begin{pmatrix} f, hh & hd, dh \\ dh, hd & dd, dd \end{pmatrix} \end{array} \tag{7}$$

Games of conflict provide a natural and well-studied representation of competitive interactions over resources [*2*,*3*,*4*]. These games, despite being idealized, provide insight into many cooperative interactions that are not adequately captured by, say, the prisoner's dilemma [*5*]. Avoiding costly conflicts is essential for cooperation, hence these games represent anti-coordination scenarios such as congestion, pollution, and provision of public goods [*6*, *7*]; these and similar games have also been used to explore division of labor [*8*] and situations with negative externalities [*9*]. Individuals that have no incentive to avoid conflict pose a substantial threat to cooperative resolutions of these strategic interaction.

The novel feature of the current model involves introducing asymmetry in conflict ability by assigning each individual a rank, where the ranking individual dominates aggressive (*hawk-hawk*) interactions and secures the contested resource. This is a departure from the baseline symmetric game of conflict (see Table A for a review of related work). In the current model, the ranking individual receives a fixed payoff f whenever conflict occurs and the outranked individual receives a payoff of 0 in a winner-take-all type of contest based on the relative standing between the two individuals.

Related Work

We review closely related work in Table A.

	Game	Coevolution[a]	Asymmetry[b]	Weighted Network[c]	Inequality[d]
This Study	*Conflict*	*Yes*	*Yes*	*Yes*	*Yes & Dynamic*
Games of Conflict on Dynamic Networks					
[10]	Conflict	Yes	Yes	Yes	No
[11]	Conflict	Yes	No	No	No
[12]	Conflict	Yes	No	No	No
Games of Conflict on Static Networks					
[2]	Conflict	No	Yes	No	No
[13]	Conflict	No	Yes	No	No
[14]	Conflict	No	Yes[e]	No	No
[15]	Conflict	No	Yes[e]	No	No
[16]	Conflict	No	No	No	Yes[f]
[17]	Conflict	No	No	No	No
[6]	Conflict	No	No	No	No
[18]	Conflict	No	No	No	No
[19]	Conflict	No	No	No	No
[20]	Conflict	No	No	No	No
[7]	Conflict	No	No	No	No
Dynamic Networks but not Games of Conflict					
[21]	Stag Hunt	Yes	No	No	No
[22]	Coordination	Yes	No	No	No
[23]	Coordination	Yes	No	No	No
[24]	PD	Yes	No	No	No
[25]	PD	Yes	No	No	No
[26]	PD	Yes	No	No	No
[27]	PD	Yes	No	No	No
[28]	PD	Yes	No	No	No
[29]	PD	Yes	No	No	No
Inequality					
[30]	Public Goods	No	No	No	Yes
[31]	Public Goods	No	No	No	Yes

Table A. Summary of most closely related work.

Note. We include only the most closely related studies that address one of the key aspects of our work: Games of conflict, dynamic networks, or inequality. Two review papers of work on dynamic networks are Gross & Blasius [32] and Perc & Szolnoki [33].

[a] This column focuses on whether the study uses networked interaction and allows partner choice. That is, network ties are allowed to coevolve with behavioral strategies.

[b] This column indicates whether or not the model comprises asymmetric strategies for hosting vs. visiting behavior, thus allowing correlated conventions as possible solution equilibria.

[c] This column indicates whether or not the model uses discrete network ties, which requires an assumption about network density (or model network density directly through a parameter).

[d] This column indicates if models includes inequality among agents such as differences in fighting ability. Both studies in this category are human-subject experiments.

[e] Their models do not distinguish asymmetry between hosting and visiting (and thus precludes the correlated equilibria as a solution) but include interesting asymmetry in the games faced by different agents, or allow actors to deploy different actions against different partners, similar to ours.

[f] Inequality is based on network connectivity (which is static) and affects the payoffs in cooperative dove-dove interactions, but not in hawk-hawk conflict.

Static game analysis

For the sake of simplicity, presume $hh = 0$ and the payoff preference ordering for a game of conflict: $hd > dd > dh > hh$. For group size N, each player has a rank $i \in \{1, 2, \ldots, N\}$. If $i < j$ then the *hawk-hawk* payoff to player i is f (and the payoff to player j is 0—the ranking individual wins the contest). If $i > j$ then *hawk-hawk* payoff to player i is $hh = 0$ (and the payoff to player j is f—the outranked individual loses the contest).

Suppose a player i is paired at random with another player j without knowledge of individual rankings. For any two players with ranks $i, j : i \neq j$ (i.e., there are no ties in rankings). Let R be the probability player i outranks partner j where

$$R = 1 - \frac{i-1}{N-1}. \tag{8}$$

Then the expected payoffs for *hawk* and *dove* depend on the probability of outranking an opponent and the probability an opponent plays *hawk*:

$$E(hawk_i) = P(hawk_j)[R \cdot f + (1-R) \cdot hh] + P(dove_j) \cdot hd, \tag{9}$$

$$E(dove_i) = P(hawk_j) \cdot dh + P(dove_j) \cdot dd. \tag{10}$$

Assuming $hh = 0$ and $P(dove_j) = 1 - P(hawk_j)$:

$$E(hawk_i) = hd + P(hawk_j)(R \cdot f - hd), \tag{11}$$

$$E(dove_i) = dd + P(hawk_j)(dh - dd). \tag{12}$$

Player i strictly prefers to play *hawk* iff $F(hawk_i) > F(dove_i)$ which is true just in case:

$$P(hawk_j) < \frac{hd - dd}{(dh - dd) - (R \cdot f - hd)}. \tag{13}$$

Or equivalently:

$$R > \frac{dh - dd + hd}{f} - \frac{(hd - dd)}{P(hawk_j) \cdot f}. \tag{14}$$

In the situation where $E(hawk_i) = E(dove_i)$ (opponents play *hawk* with a probability equal to right hand side of condition 13) then player i is indifferent between playing *hawk* or *dove*. Note that for the bottom ranked individual ($R = 0$) this condition amounts to the mixed Nash equilibrium for the background game of conflict.

Finding the dominant solvable breakpoint

Given the asymmetry created by differences in power or competitive ability, the game may be dominant solvable for some top ranking individuals. We can identify this region by plotting an indifference line as a function of the probability of encountering a ranking or outranked individual. That function is

$$P(hawk_j) = \frac{(hd-dd)}{(dh-dd)-(R\cdot f-hd)} \tag{15}$$

This is the point at which, if your opponent should play *hawk* with this probability, you are indifferent between $(hawk, dove)$ presuming $P(hawk_j) \in [0,1]$. This point will not exist for all players. Depending on the exact payoffs, only some players will have an indifference point. So, for instance, top ranked individuals will have an "indifference point" outside the unit interval, which simply means that *hawk* is their dominant strategy. If $0 < P(h_j) < 1$ then there exists a point at which, should an opponent play *hawk* with higher probability, the player prefers *dove*; and should an opponent play *hawk* with lower probability, the player prefers *hawk*. In effect, the player does not have a dominant strategy in the population. Note that the lowest ranked individual in the population has an indifference point equal to the mixed Nash equilibrium for the standard hawk-dove (where $f = hh$) game since the probability that player ever plays the alternative game is zero.

In the rank ordering there is a breakpoint (i_t) between ranking individuals that have a dominant strategy (*hawk*) and outranked individuals that have an indifference point below one ($P(hawk_j) < 1$). To solve where in the ranking the breakpoint exists set $P(hawk_j) = 1$ and solve for R (the probability i outranks j):

$$R\cdot f = dh. \tag{16}$$

Using equation 8 to substitute for R we can solve for the rank transition point i_t:

$$i_t = (1-\frac{dh}{f})(N-1)+1. \tag{17}$$

If a player's rank is better than i_t (i.e., the player's rank value is strictly less than the breakpoint i_t or $i < i_t$) then they have a strongly dominant strategy (*hawk*). In the situation where $i = i_t$ the player prefers to play *hawk* if their opponent plays *dove* but is indifferent between *hawk* and *dove* if their opponent plays *hawk*; in effect, *hawk* is a weakly dominant strategy for player i. Therefore one should play *hawk* when $i \leq i_t$. Otherwise (when $i > i_t$) the player has an indifference point that identifies when they would prefer to play *hawk* or *dove*. This is represented in Fig 2A in the main text.

Breakdown of conventions

In this strategic scenario bullies (individuals that can win any contest and can break with conventions) emerge when $f \geq dh$. This transition point does not depend on population size. The breakpoint conditions (equations 16-17) identify the threshold where top-ranked individuals may stop following correlated convention and emerge as bullies (individuals that can win any contest and can ignore the convention). Since $R = 1$ for the top-ranked individual the condition for this transition is $f \geq dh$. This marks the point where the top ranked individual faces a dominant solvable game. When $hh < f < dh$ even top-ranked individuals prefer to adhere to the correlated equilibrium convention for the hawk-dove game. In cases where $f < dh$ the rank transition i_t is negative and therefore no one in the population faces a dominant

solvable game. Once $f \geq dh$ then ranking individuals near the top of the hierarchy will stop adhering to the convention, though when f is close to dh most of the population continues to adhere to the convention. This transition does not depend on population size.

Is there a point when the convention becomes impossible to sustain? When f reaches a high value there looks to be a point where the convention starts to breakdown across the population and simulations show that conventions tend to go extinct. Analytically, there is a useful comparison to assess this question: when does does the expected payoff of following the convention drop below the expected payoff for pure doves? Consider the $host - guest$ convention where player i plays *dove* as host and *hawk* as visitor. When is it the case that $E(dove_i) > E(para_i)$? To simplify the comparison, presume players can encounter either pure strategies ($hawk, dove$) or *para* (play *dove* at home and *hawk* away).

The expected payoffs depend on rank, opponent strategy, and whether the player is host or visitor. Note that both *dove* and *para* play the same strategies as host. The key part of the comparison involves how they do when visiting. Assuming individuals interact once as host and once as visitor with a randomly selected individual in the population then whether $E(dove_i) > E(para_i)$ depends on a comparison of expected payoffs when visiting:

$$\begin{aligned} & P(para_j) \cdot dd + P(dove_j) \cdot dd + P(hawk_j) \cdot dh > \\ & P(para_j) \cdot hd + P(dove_j) \cdot hd + P(hawk_j) \cdot R \cdot f. \end{aligned} \tag{18}$$

Given that $P(para_j) + P(dove_j) + P(hawk_j) = 1$ and $P(para_j) + P(dove_j) = 1 - P(hawk_j)$ this inequality simplifies to:

$$P(hawk_j) > \frac{hd - dd}{(dh - dd) - (R \cdot f - hd)}. \tag{19}$$

Note that this is a version of the decision scenario described by condition 13 just reversed to consider when a player prefers to play *dove* rather than *hawk*. However, when f is high several individuals will confront a dominant solvable game where they always play *hawk* and therefore $P(hawk_j) = 1$ when encountering these individuals. If there are sufficient numbers of these pure *hawk* individuals, visitors will always prefer *dove* to *para* and the convention becomes impossible to sustain. For instance, consider the case where $N = 20$ and the game payoffs are $hh = 0, dh = 0.4, dd = 0.6, hd = 1$. According to the mixed Nash equilibrium for these payoffs in a standard game of conflict, a player (strictly) prefers to play *dove* when $P(hawk_j) > 0.5$. In a mixed population of pure *hawk* and *dove*, a player will prefer to be a *dove* when *hawk* makes up more than half the population. For the $N = 20$ case, half the population has faces a dominant solvable confrontation (i.e., $i_t = 10$), and therefore play as pure *hawk*, when $f = 0.76$. At this point, it does not matter what the rest of the population is doing because the chances of encountering a hawk (assuming random interactions) are sufficiently high that a new member of the population prefers to play *dove* regardless. The exact threshold can be determined by assessing the expected $P(hawk_j)$ of possible opponents weighted by the probability of encountering different individual strategy profiles. This will depend on the payoffs of the game, the network structure, an individual's ranking, and the population size. The learning dynamics will also affect how populations tend to behave (i.e., whether conventions go extinct) close to the threshold.

Static networks

As individuals learn their own rank through reinforcement learning, they move through the space of game strategies (Fig A). On static networks and high f–cases in which the correlated convention breaks–out-ranked individuals adopt

defensive strategies playing dove both at home and away, while ranking individuals adopt aggressive strategies playing hawk in both cases.

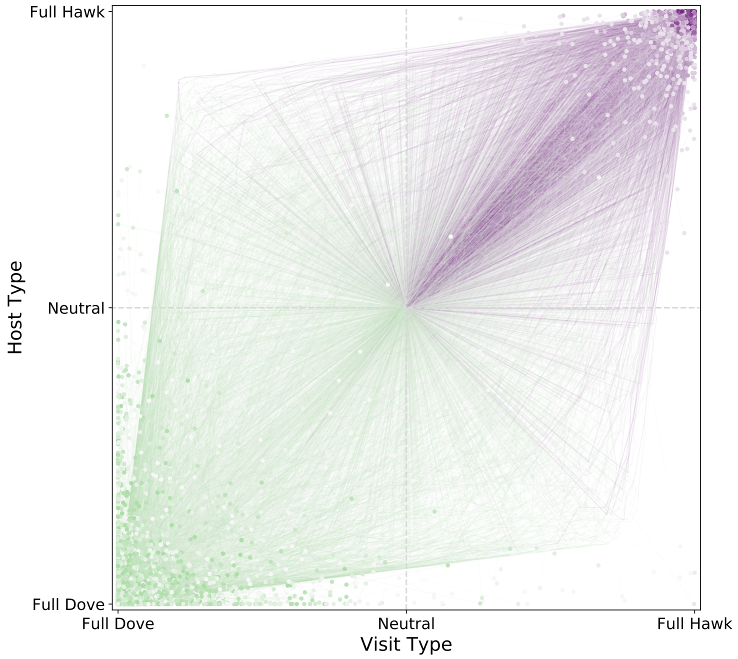

Fig A. Evolution of strategies shown as trajectories over time. Individuals of all ranks start at center at $t = 0$ with 50:50 hawk:dove. Ranking individuals learn hawk-hawk and move to the top-right. Outranked individuals learn dove-dove and move to bottom-left ($dh = 0.4; dd = 0.6; f = 0.9$).

Dynamic networks

Expanding on the network evolution shown in the main text which show different seeds aggregated by f, we show raw, unaggregated data in Fig B.

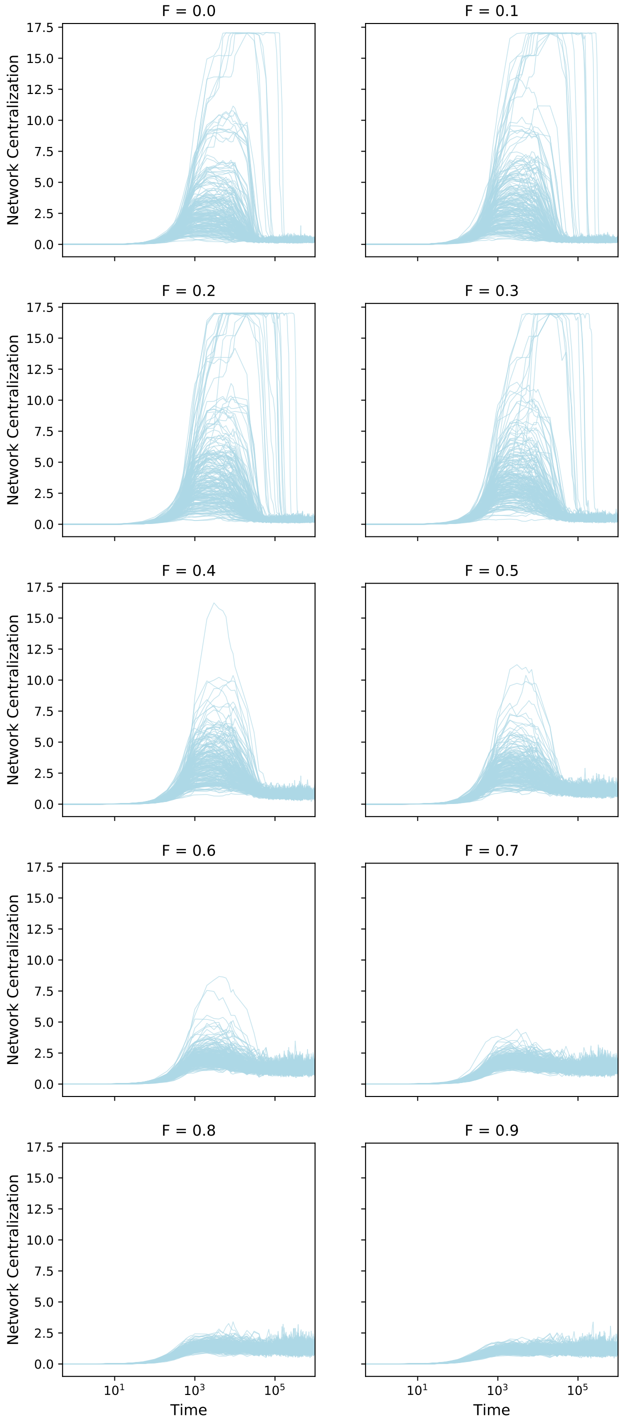

Fig B. Network centralization over time for each seed, grouped by f value. As reference, an ideal star network has *Network Centralization* $= 17.95$. Stars disappear for $f \geq 0.4$.

Partner choice and dynamic ranks

As we have seen, cycles start to emerge at the threshold $f \geq dh$ but do all individuals cycle through ranks or just some? To explore this question, we analyze the percentage of time that each individual spends in each rank (Fig C). We find that each individuals cycle through ranks and spends close to an equal amount of time in each rank (5% in our the case of our standard population size of 20). However, we notice one interesting pattern. Occupying the top rank and the bottom rank are somewhat correlated: only in the bottom rank can an individual accumulate enough payoffs to make it all the way to the top of they hierarchy. Simply being near the bottom is not enough to make it all the way to top. So while some individuals oscillate between the extremes, others spend most of their time oscillating only between middle ranks.

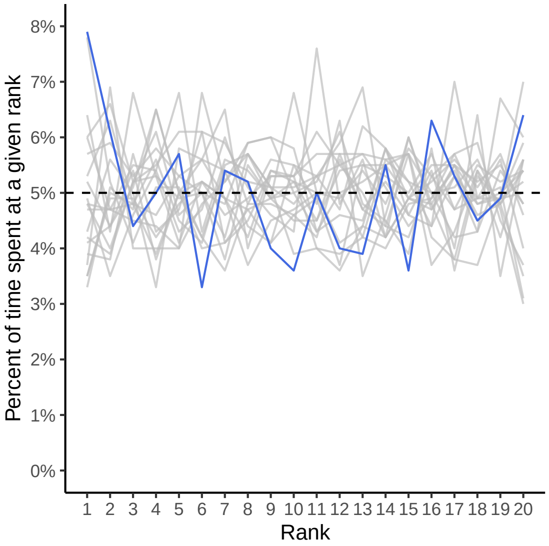

Fig C. All individuals cycle through ranks and spend close to an equal amount of time in each rank. Each line shows one individual from one example seed of a population of 20 ($f = 0.8$). Individual with the highest standard deviation of ranks highlighted in blue. Dashed line shows expected time in each rank for population of (5% in the case of population of 20).

The ability of individuals to accumulate payoffs is crucial to the cycles observed in the central model. If *dove*-playing individuals were unable to accumulate payoffs, perhaps because losses were significantly costly or negative, cycles would not emerge. To illustrate this we can introduce an artificial modification to the model (artificial because Roth-Erev reinforcement learning cannot accommodate negative payoffs without being modified). We explore rank updating dynamics with a rule that mimics negative *hh* and *dh* payoffs by subtracting a constant c per interaction for assessing rank changes only (the reinforcement of strategy and network weights proceeded as usual with the Roth-Erev updating). When $c = dh$ this effectively treats a *dh* payoff as zero and *hh* as negative. When $c > dh$, both *dh* and *hh* are negative. When we adopt this rule for rank updating we do not see the cycling behavior because individuals cannot accumulate payoffs from playing dove as host to enable a rise in the ranks. Instead, we see noisy rank changes across the length of simulation. This noise occurs because few individuals are receiving positive per-interaction payoffs (i.e., *hd* or *dd*) so rank changes for many individuals become a random walk. These individuals are unable to learn their effective ranks and so play more noisily towards highly ranked individuals which introduces additional fluctuations in rank. However, the correlated convention (playing *hawk* away and *dove* at home) still emerges in these cases with one small difference. Some (top-ranked) individuals are very noisy in their strategy choice as hosts, often oscillating between *dove* and *hawk* host plays. Visitor strategy choice quickly converges on *hawk* for all individuals.

Timescale comparison

Partner choice and static power asymmetries. First, we investigate the effect of different learning speed timescales in the case of static ranks. We change the timescale of network updating relative to strategy updating by adjusting the payoffs used to update network weights (see Methods). Our results are robust to differences in timescales of network learning and strategy learning (Fig D). When power asymmetry is low, faster network learning (relative to strategy learning) leads to higher proportion of paradoxical interactions at the final time step, and slower network learning leads to lower proportion of paradoxical interactions. However, paradoxical interactions are still a significant proportion overall (about 90%). When power asymmetry is high, network updating speed does not have a discernable effect on the resulting proportion of paradoxical interactions.

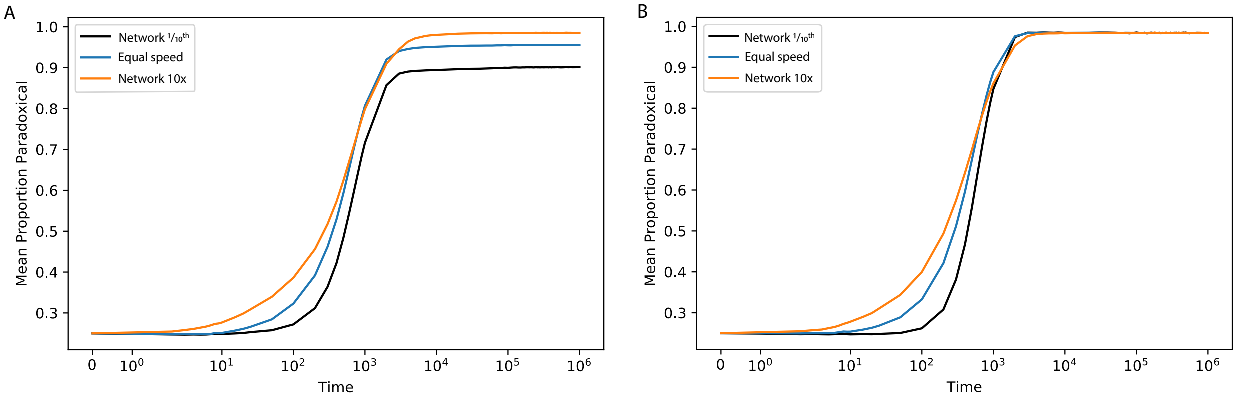

Fig D. Comparison of relative learning speeds. A, $f = 0.2$. **B**, $f = 0.7$. (Both panels use $dh = 0.4; dd = 0.6$).

We also analyze the evolution of pure hawk nodes across different relative learning speeds (Fig fig:supNetworkLearningSpeeds2). For the purpose of this analysis, we defined nodes as "pure hawk" if they had least 0.8 hawk strategy weight for both visitor and host (the exact specification does not change the results substantively and is made only for illustration purposes). While the convention breaks for random interaction (static network; black line $v = 0.0$), the convention continues to survive even when networks evolve very slowly, or faster.

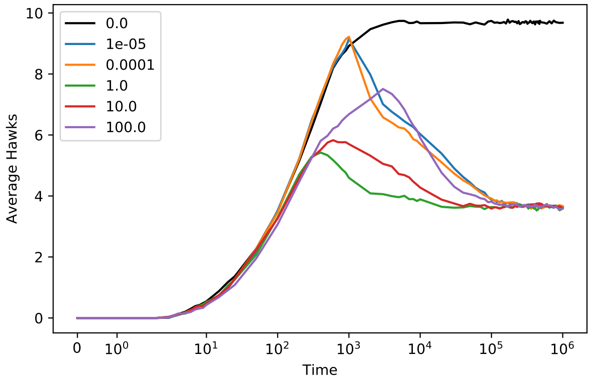

Fig E. Even slow updating of partner choice allows the convention to survive. Without network updating ($v = 0.0$), the convention breaks but survives with both faster and slower network learning speeds ($dh = 0.4; dd = 0.6; f = 0.8$).

Partner choice and dynamic power asymmetries. Next, we investigate the effect of varying discount rates in the dynamic ranks case. Varying the discount for network learning, but not strategy learning, changes how fast network ties can be revised after changes in strategy relative to strategic learning. The lower the discount rate (δ) the longer it takes for network ties to be revised. We examine discount rates of 0.001 and 0.0001 in comparison to the default rate of 0.01, meaning network learning speed is $^1/_{10}{}^{th}$ and $^1/_{100}{}^{th}$ of the strategy learning speed in this respect. In both cases, the observed cycles take much longer. (Fig F). As cycle length increases, some cycles may become longer than the 1 million time steps of our simulations. This is the case in several individual simulations as noted in the figure caption.

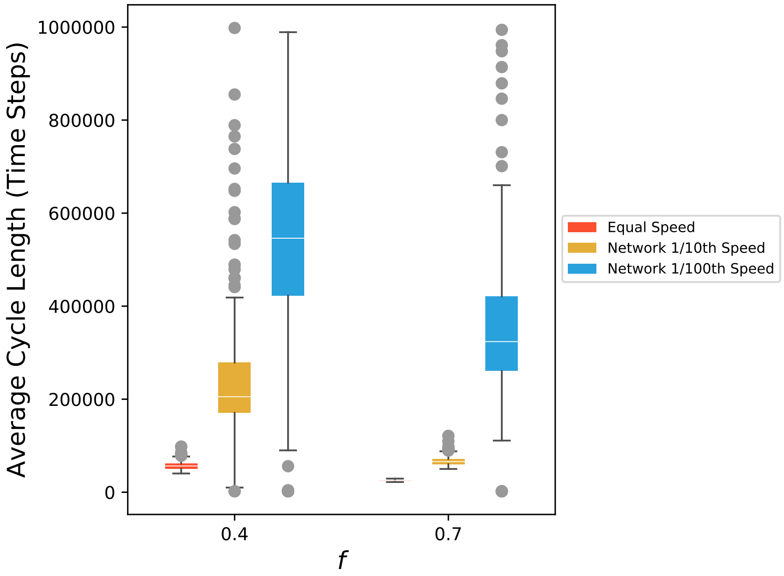

Fig F. Cycle length naturally depends on speed of network learning. In some cases, cycle lengths exceed the 1 million time steps of our simulations. For power asymmetry of $f = 0.4$ this is the case in 2.5% of simulations at $1/10$ network speed and 25% of simulations at $1/100$ network speed; for power asymmetry of $f = 0.7$ 1% of simulations exceed the time limit at $1/100$ network speed ($dh = 0.4; dd = 0.6$).

Inequality

In this section we provide detailed results of total payoffs and inequality across the full range of f-values. We focus on the contrast between static and dynamic networks, and between static and dynamic ranks. Across all regimes, cumulative payoffs are highest in the case of f below the critical value of dh: in this regime, ranks effectively do not matter and thus there are no bullies that cause "friction" compared to the correlated convention (Fig G). With $f > dh$ bullies start to disrupt the correlated convention and cumulative payoffs dip. The cases with static networks are identical as individuals do not change their rank if they cannot be isolated. In those two cases where individuals cannot avoid powerful bullies and the correlated convention breaks payoffs dip the most, reaching the lowest payoff at $f = 0.5$. After that, cumulative payoffs increase slightly as the increasingly large f payoff earned by bullies starts to offset some of the friction caused by the broken correlated convention.

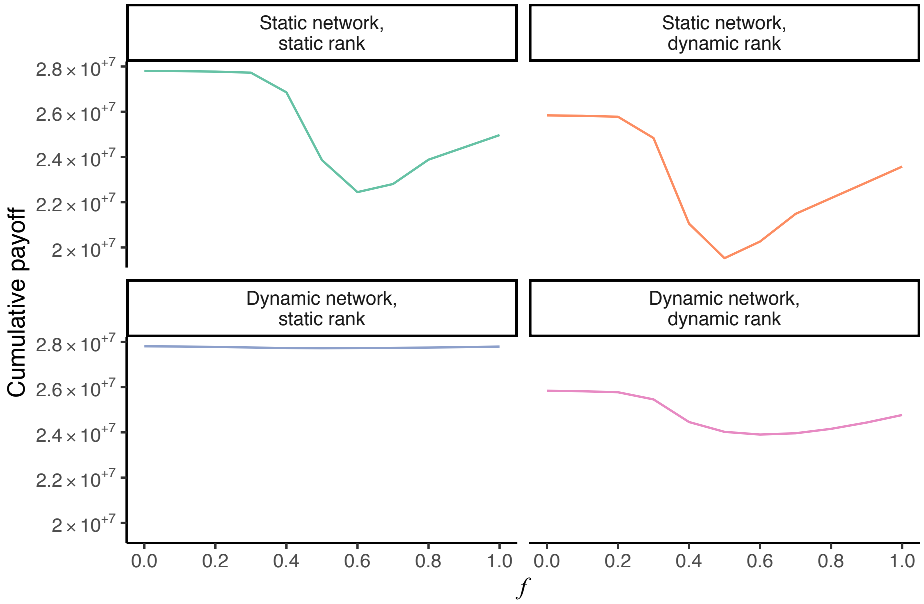

Fig G. Total payoffs by f.

Inequality is lowest in the case of dynamic networks with dynamic ranks as the existence of cycles allow each individual to spend equal time at the top of the hierarchy (Fig H). Inequality is highest in the static network cases and is highest in the case of more extreme asymmetry (higher f). In the case of dynamic networks and static ranks, inequality increases with increasing asymmetry, but then decreases with very high asymmetry as fewer top individuals break away from the correlated convention to adopt aggressive pure hawk strategies as shown in Fig 3A in the main text.

How do payoffs differ across individuals of different ranks? We show results using $f = 0.6$ as a canonical case in which power asymmetry is large enough for bullies to emerge, but not large enough for the convention to break completely (Fig I). On static networks, high-ranking individuals become bullies and earn higher payoffs than individuals who are part of the cooperative convention (who all earn the same payoffs). In static networks, individuals cannot be isolated, so ranks never change even when they are allowed to change in principle. With partner choice and static ranks, the pattern reverses: high-ranking bullies get isolated while low-ranking cooperators attract more visitors and thus earn higher payoffs. As there is some variation in the network structures that emerge in this case, there is variation in how many visitors low-ranked individuals attract and hence their payoffs can vary substantially more than in all other cases. The overall pattern is clear: the lowest ranked individual earns the highest payoffs, followed by the second-lowest ranked and so on. Finally, on dynamic network with dynamic ranks, all individuals move through ranks in cycles,

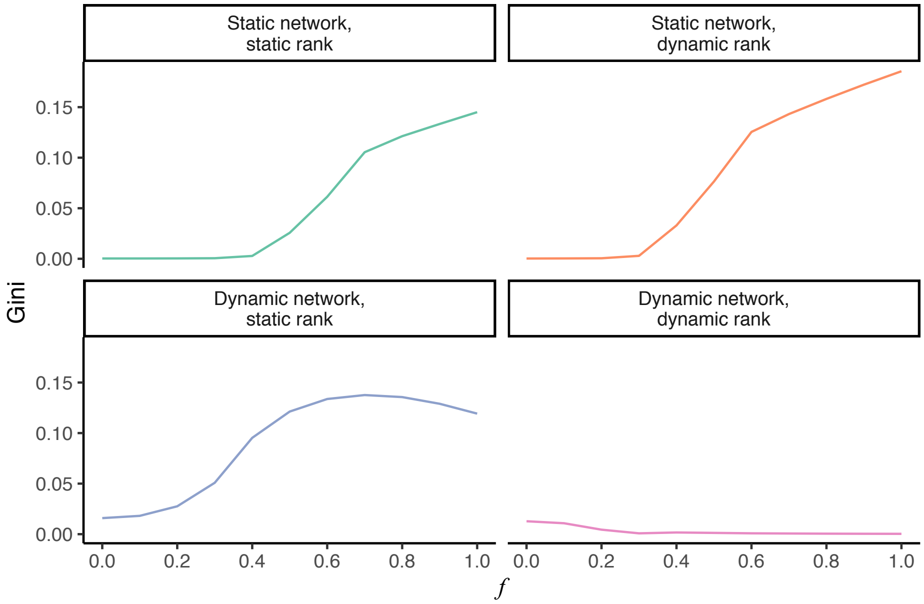

Fig H. Gini by f.

spending about equal time in each rank (as shown above in Fig C). As a result, all individuals earn similar payoffs, no matter their initial rank.

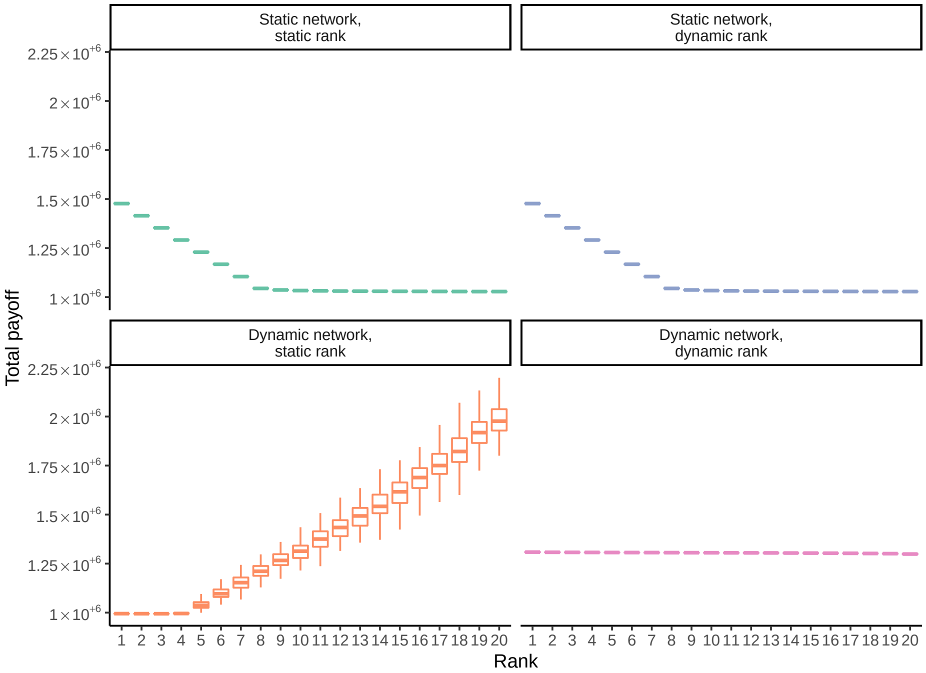

Fig I. Total payoffs by initial rank contrasting all four combinations of our model. ($f = 0.6$; box-plot shows distribution of payoffs at each rank across 200 seeds)

Population Size

We find all our results are robust across different population sizes. For our analysis we define an individual as a "pure hawk" if the likelihood of playing hawk both at home and as visitor is greater than 0.8. We make this definition purely for convenience so that we can show proportions of agents by "type". The substantive result of the overall pattern of the curves shown in Fig 3B in the main text does not critically depend on the likelihood threshold and looks similar for higher (e.g., 0.9) or lower thresholds (e.g., 0.6). All simulations with population size up to 100 are 200 seeds, population 200 is 50 seeds, and population 500 is 20 seeds.

The presence of cycles in the model with network learning and dynamic ranks does not depend on population size. Fig J shows distribution of cycle lengths across f for different population sizes.

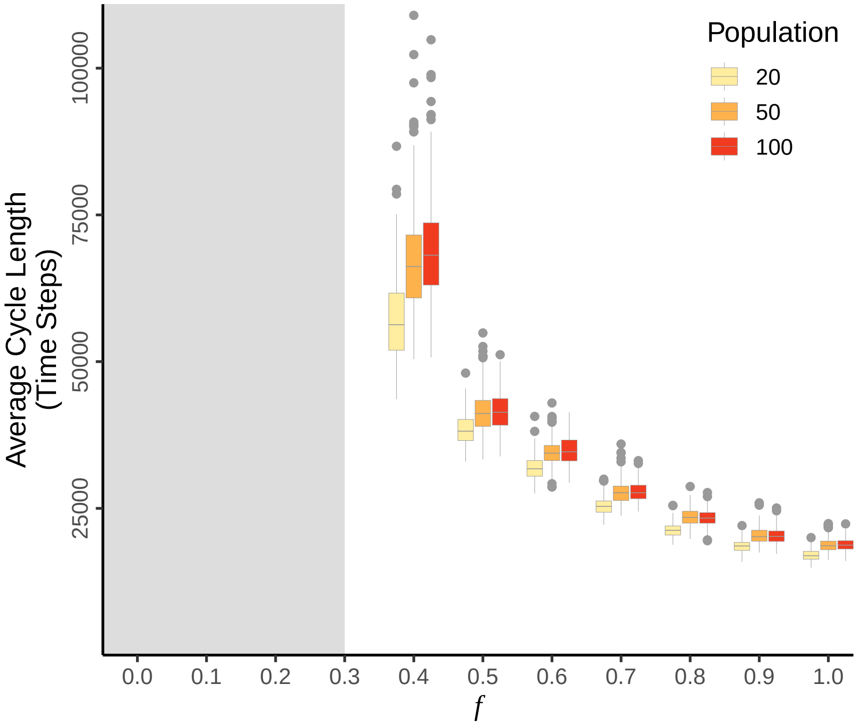

Fig J. Cycle lengths are somewhat longer in larger populations. The overall pattern of shorter cycles with higher power asymmetry remains robust.

References

1. Weibull, J. W. *Evolutionary Game Theory* (MIT Press, 1997).
2. Smith, J. M. & Parker, G. A. The logic of asymmetric contests. *Animal Behav.* **24**, 159–175 (1976).
3. Smith, J. M. *Evolution and the Theory of Games* (Cambridge University Press, 1982).
4. Grafen, A. The logic of divisively asymmetric contests: respect for ownership and the desperado effect. *Animal Behav.* **35**, 462–467 (1987).
5. Bardhan, P. Irrigation and cooperation: An empirical analysis of 48 irrigation communities in south india. *Econ. Dev. cultural change* **48**, 847–865 (2000).
6. Doebeli, M. & Hauert, C. Models of cooperation based on the prisoner's dilemma and the snowdrift game. *Ecol. Lett.* **8**, 748–766 (2005).

7. Bramoullé, Y. Anti-coordination and social interactions. *Games Econ. Behav.* **58**, 30–49 (2007).
8. O'Connor, C. *The Origins of Unfairness: Social Categories and Cultural Evolution* (Oxford University Press, USA, 2019).
9. DeCanio, S. J. & Fremstad, A. Game theory and climate diplomacy. *Ecol. Econ.* **85**, 177–187 (2013).
10. Foley, M., Forber, P., Smead, R. & Riedl, C. Conflict and convention in dynamic networks. *J. Royal Soc. Interface* **15**, 20170835 (2018).
11. Pacheco, J. M., Traulsen, A. & Nowak, M. A. Coevolution of strategy and structure in complex networks with dynamical linking. *Phys. Rev. Lett.* **97**, 258103 (2006).
12. Francisco C. Santos, T. L., Jorge M. Pacheco. Cooperation prevails when individuals adjust their social ties. *PLoS Comput. Biol.* **2**, e140 (2006).
13. Mesterton-Gibbons, M. & Sherratt, T. N. Bourgeois versus anti-bourgeois: a model of infinite regress. *Animal Behav.* **89**, 171–183 (2014).
14. McAvoy, A. & Hauert, C. Asymmetric evolutionary games. *PLoS Comput. Biol.* **11**, e1004349 (2015).
15. Tsvetkova, M. & Buskens, V. Coordination on egalitarian networks from asymmetric relations in a social game of chicken. *Adv. Complex Syst.* **16**, 1350005 (2013).
16. Du, W.-B., Cao, X.-B., Hu, M.-B. & Wang, W.-X. Asymmetric cost in snowdrift game on scale-free networks. *EPL (Europhysics Lett.* **87**, 60004 (2009).
17. Macy, M. W. & Flache, A. Learning dynamics in social dilemmas. *Proc. Natl. Acad. Sci.* **99**, 7229–7236 (2002).
18. Kokko, H., López-Spulcre, A. & Morrell, L. J. From hawks and doves to self-consistent games of territorial behavior. *The Am. Nat.* **167**, 901–912 (2006).
19. Wang, W.-X., Ren, J., Chen, G. & Wang, B.-H. Memory-based snowdrift game on networks. *Phys. Rev. E* **74**, 056113 (2006).
20. Zhong, L.-X., Zheng, D.-F., Zheng, B., Xu, C. & Hui, P. Networking effects on cooperation in evolutionary snowdrift game. *EPL (Europhysics Lett.* **76**, 724 (2006).
21. Skyrms, B. & Pemantle, R. A dynamic model of social network formation. *Proc. Natl. Acad. Sci.* **97**, 9340–9346 (2000).
22. Jackson, M. O. & Watts, A. On the formation of interaction networks in social coordination games. *Games Econ. Behav.* **41**, 265–291 (2002).
23. Goyal, S. & Vega-Redondo, F. Network formation and social coordination. *Games Econ. Behav.* **50**, 178–207 (2005).
24. Fu, F., Hauert, C., Nowak, M. A. & Wang, L. Reputation-based partner choice promotes cooperation in social networks. *Phys. Rev. E* **78**, 026117 (2008).
25. Fu, F., Wu, T. & Wang, L. Partner switching stabilizes cooperation in coevolutionary prisoner's dilemma. *Phys. Rev. E* **79**, 036101 (2009).
26. Van Segbroeck, S., Santos, F. C., Nowé, A., Pacheco, J. M. & Lenaerts, T. The evolution of prompt reaction to adverse ties. *BMC Evol. Biol.* **8**, 287 (2008).
27. Van Segbroeck, S., Santos, F. C., Lenaerts, T. & Pacheco, J. M. Reacting differently to adverse ties promotes cooperation in social networks. *Phys. Rev. Lett.* **102**, 058105 (2009).

28. Wu, B., Zhou, D., Fu, F., Luo, Q. & Wang, A., L.AND Traulsen. Evolution of cooperation on stochastic dynamical networks. *PloS One* **5**, e11187 (2010).

29. Pinheiro, F. L., Santos, F. C. & Pacheco, J. M. Linking individual and collective behavior in adaptive social networks. *Phys. Rev. Lett.* **116**, 128702 (2016).

30. Hauser, O. P., Kraft-Todd, G. T., Rand, D. G., Nowak, M. A. & Norton, M. I. Invisible inequality leads to punishing the poor and rewarding the rich. *Behav. Public Policy* 1–21 (2019).

31. Hauser, O. P., Hilbe, C., Chatterjee, K. & Nowak, M. A. Social dilemmas among unequals. *Nature* **572**, 524–527 (2019).

32. Gross, T. & Blasius, B. Adaptive coevolutionary networks: a review. *J. Royal Soc. Interface* **5**, 259–271 (2008).

33. Perc, M. & Szolnoki, A. Coevolutionary games—a mini review. *BioSystems* **99**, 109–125 (2010).
