# Supplementary material for: Avoiding the bullies: The resilience of cooperation among unequals
Source: PLoS Comput Biol. 2021 Apr 7;17(4):e1008847. doi: 10.1371/journal.pcbi.1008847 (PMC8055019; doi:10.1371/journal.pcbi.1008847)
Supplement: Attachment — Submitted filename: foleyreview.pdf [file pcbi.1008847.s002.pdf]

Referee Report for Foley et al
Avoiding the bullies: Reslience of cooperation among unequals

The authors consider a series of models for asymmetric games of conflict (i.e. a family of two-playe, two-strategy Hawk-Dove/Snowdrift games) played on networks in which players update their strategies and, in some models, their weights for network interactions using a reinforcement learning alogorithm. Connecting to ideas on dominance hierarchies from the animal behavior literature, individuals with higher rank receive a greater payoff in hawk-hawk interactions than their lower-ranked opponents, which models the higher-ranked individuals' enhanced ability to win in a fight with an outranked peer. Of particular interest are cases in which the ranking individual is sufficiently better at fighting such that the hawk/defection action becomes their dominant strategy, breaking down correlated-conventions for sustaining the mixed equilibrium in the population. By allowing the possibility of partner choice, the authors show that individuals will choose to avoid interacting with high-rank "bullies" that always defect, and, with fixed rankings, this allows a subset of the population to sustain the cooperative convention that the population supports for symmetric Hawk-Dove games. By further allowing the individual rankings to depend on discounted cumulative payoff, the authors further find cycling behavior in both rankings and cooperative behavior. In this regime, low-ranked individuals can attract visitors by cooperating, and then their increased interactions allow them to rise the population ranks until defection becomes their dominant strategy, and they become the bullies that their peers choose to avoid, causing their ranking to drop and starting the cycle anew.

I think that this paper is an interesting and relevant contribution to the literature on the evolution of cooperation for games on networks, particularly in connection to work on dominance hierarchies in animal populations. The result on the cycling behavior in the model with partner choice and dynamical ranking based on discounted cumulative payoff is particularly interesting, and provides an additional qualitative behavior for promoting cooperative behavior with adaptive networks. I believe that the results of this paper are of appropriate technical quality and biological interest to be published in *PLoS Computational Biology*. However, I also think that more work required to improve the exposition of the paper, as well as to contextualize how the assumptions of the authors' models impact the long-time strategic and network dynamics.

Here are some major comments that I believe can help to improve the paper.

- An assumption that might help to drive the possibility of cycling behavior in the dynamical ranking cases appears to be that the dove-hawk payoff $dh > 0$, so that low-ranked individuals can accumulate high total payoffs by playing a lot of games with higher-ranked opponents who defect. Even though dh is third-best possible payoff, when it is positive, a large number of dh payoffs can produce more total payoff than a small number of dd or hd payoffs. Is the same kind of cycling behavior possible if $hh < dh < 0$? It may be the case this this is possible if what is primarily driving the cycles is that low-ranking individuals can receive many dd payoffs by interaction with other lower-ranked individuals.
- On a similar note, how do the dynamics of this model depend on the relative value of the payoffs $2(dd)$ received from the interaction of two doves and $dh + hd$ received from the interaction of a hawk and a dove? Does this change the social welfare of the long-time outcome, or alter the ability to maintain the coordinating-convention rather than spliting into pure hawk and pure dove behaviors.
- In addition, I have several comments on the presentation of the payoff matrix and for the analysis of the static game.

- I believe the payoff matrix (in table 1) should be presented much earlier in the paper, so that references to the payoff parameters in the main text are more clearly understood without having to first read the appendix.
- I don't think the use of the symbol $\vee$ appropriately conveys the asymmetry of payoffs for players with different ranks. If, without loss of generality, we consider the interaction with players whose ranks satisfy $i < j$, one can present the payoffs to an i (row) and j (column) player through the following asymmetric payoff matrix

$$\begin{array}{c} \\ \text{hawk}_i \\ \text{dove}_j \end{array} \begin{array}{c} \begin{array}{cc} \text{hawk}_j & \text{dove}_j \end{array} \\ \begin{pmatrix} f, hh & hd, dh \\ dh, hd & dd, dd \end{pmatrix} \end{array}. \tag{1}$$

  - ∗ Formulations of similar asymmetric games in which either both players face a Hawk-Dove game or in which one player faces a Prisoners' Dilemma and the other a Hawk-Dove game are studied in the paper of McAvoy and Hauert [1] or by Hall et al [2].
- If, in addition, the assumption that $hh = 0$ is delayed for the static game analysis, one can derive more general conditions for the relative ranking of individuals required for an individual to have dominance solvability as a pure hawk player. In particular, the condition of Equation 9 becomes

$$P(\text{hawk}_j) < \frac{hd - dd}{hh - dd - (Rf + (1-r)hh - dh)}, \tag{2}$$

  and then individuals who outrank the fraction R of the population should play pure hawk when

$$R > R^* = \frac{dh - hh}{f - hh}. \tag{3}$$

  Therefore the threshold dominance probability $R^* < 1$ when $dh < f$, agreement with the special case in which $hh = 0$. In particular, the fact that this is true even when payoff values are potentially negative provides some more motivation for understanding whether the cycling behavior is still possible if $dh < 0$ and whether the dynamics seen under partner choice can be achieved through high total payoff or high average payoff across interactions.

- The mechanism governing the partner choice and the subsequent maintenance of the coordinating convention with static rankings or cyclic behavior with dynamical ranking differs from the mechanisms often studied on the evolution of cooperation in adaptive networks. Mechanisms like active linking [3] or assortative interactions [4] tend to help form clusters of cooperators who attain higher average payoffs than defectors who end up interacting with other defectors. It could for the authors to provide more description of how the behaviors in this model differ from how previous work has studied evolution of cooperation on adaptive networks for both Prisoners' Dilemma games and games of conflict.

- It may also be relevant to discuss the extent to which certain behaviors require the host-guest paradigm of interactions. For the static analysis, the two main results about of high-ranking individuals facing a Prisoners' Dilemma problem for sufficiently large f and that low-ranking individuals then see all-dove as the best-response against a population with many all-hawk individuals can be realized through a single type of interaction. In addition, the cycling behavior could possibly still arise under a similar partner choice scheme where each player chooses one opponent

and plays one strategy against their opponent of choice as well as individuals that choose them for interactions. For the model with partner choice and static rankings, how would allowing only a single kind of interaction affect the long-term levels of cooperation (as the correlated-convention is no longer possible). If any of the behaviors do or do not require the host-guest framework, it would be helpful to discuss why this is the case.

  – Correspondingly, the discussion on page 20 for the breakdown of the correlated-convention can probably be simplified. Equations 14 and 15 seem potentially redundant given the information discussed before Equation 16.

I also have the following minor comments that can help to improve the clarity of the paper and to help emphasize the key results and the relationship to the underlying assumptions.

- For the purpose of illustrating the competition over shared resources that typify games of conflict, I think it may be useful to present some parametrizations of the asymmetric payoff matrix (either when you introduce the general payoff matrix, or in the methods section or an appendix). One paramterization of potential interest for this paper would be the payoff matrix considered by Hall et al in models of repeated snowdrift games in which individuals have unequal ability to win a fight, yielding asymmetric payoff from the hawk-hawk interaction [2].
- In the methods section, it would be useful to understand how the weights for network interactions are updated based on the payoffs from previous interactions. It would probably suffice to include analogues of Equations 2 and 3 that describe how strategies depend on payoff.
- In several places, the term fitness function is used to describe the expected payoff for playing strategies like hawk or para. The term fitness probably should not be used here, because strategy updates depend on reinforcement learning rather than social imitation or birth-death events, and the term expected payoff is actually what is more directly being computed in the static analysis anyway.
- Another potentially relevant reference for games of conflict with partner choice is a paper by Tsvetkova and Buskens which considers models and experiments on chicken games in which individuals can choose their network neighbors [5].
- There is a fair bit of repetition in the paper. I particularly notice several iterations of the explanation of the cycling behavior (which potentially provides important intuition) and multiple observations about how the threshold behavior $f > dh$ does not depend on population size. For the latter comment, it is useful to know that the dynamics are not strongly dependent on population, but is also probably not necessary to overemphasize this overation after considering that ranking individuals see hawk as a dominant strategy precisely when $f > dh$, setting into motion the need for others to respond to the all-hawk actions of high-ranking individuals.
- For Figure 14, the axis labels are missing. The plot is understandable from the description above, but it may still help to revise the figure.

References

[1] A. McAvoy and C. Hauert, "Asymmetric evolutionary games," *PLoS Comput Biol*, vol. 11, no. 8, p. e1004349, 2015.

[2] C. L. Hall, M. A. Porter, and M. S. Dawkins, "Dominance, sharing, and assessment in an iterated hawk–dove game," *Journal of theoretical biology*, vol. 493, p. 110101, 2020.

[3] J. M. Pacheco, A. Traulsen, and M. A. Nowak, "Active linking in evolutionary games," *Journal of theoretical biology*, vol. 243, no. 3, pp. 437–443, 2006.

[4] T. C. Bergstrom, "The algebra of assortative encounters and the evolution of cooperation," *International Game Theory Review*, vol. 5, no. 03, pp. 211–228, 2003.

[5] M. Tsvetkova and V. Buskens, "Coordination on egalitarian networks from asymmetric relations in a social game of chicken," *Advances in Complex Systems*, vol. 16, no. 01, p. 1350005, 2013.
