# Supplementary material for: Avoiding the bullies: The resilience of cooperation among unequals
Source: PLoS Comput Biol. 2021 Apr 7;17(4):e1008847. doi: 10.1371/journal.pcbi.1008847 (PMC8055019; doi:10.1371/journal.pcbi.1008847)
Supplement: Attachment — Submitted filename: Review_PLosCB_Xin.pdf [file pcbi.1008847.s003.pdf]

In this paper, the authors aim to study the evolution of cooperation behavior (egalitarian norms/conventions) in the presence of dominant bullies (power asymmetry) using a theoretical framework of evolutionary game theory, in this case, a generalized hawk-dove game of conflict. They find the cooperative conventions can always be maintained if the system allows partner choice, letting individuals change their interaction partners via a simple reinforcement learning process, whereas such conventions would break down if individuals cannot choose their partner freely (such as the random interaction case). Further, they introduce dynamic power asymmetries where individual's rank depends on the cumulative payoffs and find the emergence of evolutionary cycles of coupled network-strategy-rank changes. The main innovation lies on the incorporation of both power asymmetry and partner choice into games of conflict, and the authors have explicitly demonstrated the nonnegligible power of these two mechanisms in persisting cooperation among unequals.

Overall, I have enjoyed reading this clearly written manuscript, with well-established motivations in an informative introduction and solid results provided by both the main text and the supplementary information. The mathematical derivations are sound and confirmed by sufficient agent-based simulations. The topic is important and attractive. And the model shows a rich set of evolutionary behaviors, some of which are counterintuitive and indeed interesting. In particular, I like the in-depth Discussion that explicitly demonstrates the important insights from the model. I believe this work provides a valuable contribution for the study of cooperation behaviors and will arouse great attention in many fields. Thereby, I am happy to recommend the acceptance of this work in its current form.

Meanwhile, I would like to provide some minor suggestions that the authors may consider for a further improvement:

(1) The first two sentences in Introduction are exactly the same as in Abstract. It's better to use a different expression.

(2) The resolution of Fig.1 b is poor in the current version. Also, it's better to add what the node size represents in the caption of Fig.1 b.

(3) I find the non-monotonous phenomenon in Fig 3 a and b where the highest proportion of pure hawks emerges at intermediate values of f is interesting and I notice that the authors have tried to provide an intuitive explanation (Line 220-221, 225-227). However, I feel that it can be better explained as a combined effect of both avoiding conflicts with ranking individuals and avoiding less benefits from hawk-dove interactions with lower-ranked individuals. The latter is caused by partner choice: lower-ranked individuals would reduce visits to a higher-ranked Hawk host.

(4) some minor mistakes:

Line 18: is dangerous work -- is a dangerous work

Line 76: in that that -- in which

Line 226: at least at least -- at least

Line 615: when does does – when does
