# Supplementary material for: Avoiding the bullies: The resilience of cooperation among unequals
Source: PLoS Comput Biol. 2021 Apr 7;17(4):e1008847. doi: 10.1371/journal.pcbi.1008847 (PMC8055019; doi:10.1371/journal.pcbi.1008847)
Supplement: Attachment — Submitted filename: 02-RESPONSE.pdf [file pcbi.1008847.s004.pdf]

Changes in this Version

Thank you for inviting us to submit a revised version of our manuscript. We appreciate the comments we received from the three reviewers assigned to our work. We were excited to hear that all reviewers liked the paper and thought it was interesting and made an important contribution to the literature on the evolution of cooperation for games on networks. As requested, we made several revisions to the manuscript and responded to each review comment point-by-point below. We have submitted two versions of the manuscript: one “final” and one that highlights all changes in the manuscript using track-changes. The reviewers made excellent constructive suggestions and we have followed their advice. The revised version of the paper includes the following key changes:

1. Added new results regarding differences in learning speed to Section *Learning speed comparison* in the SI and discuss them in the main manuscript.
2. Created new, revised Fig.1 to be more descriptive of the updating dynamics and introduce the payoff matrix earlier in the text.
3. Added clarification and more nuanced discussion of positive payoffs in Roth-Erev reinforcement learning framework.
4. Several other smaller edits to address the reviewers concerns such as removed the term “fitness”, provided a definition of “convention”, equations for network updating, and a better explanation for why cycles emerge.
5. Expanded discussion and incorporated references suggested by the reviewers to enhance the contribution of our work.
6. Made careful revisions to the entire manuscript to (a) fix typos and grammar, (b) improve structure, (c) remove repetition, and (d) improve overall readability.

Response to Reviewer 1

R#1-1. “An assumption that might help to drive the possibility of cycling behavior in the dynamical ranking cases appears to be that the dove-hawk payoff dh > 0, so that low-ranked individuals can accumulate high total payoffs by playing a lot of games with higher-ranked opponents who defect. Even though dh is third-best possible payoff, when it is positive, a large number of dh payoffs can produce more total payoff than a small number of dd or hd payoffs. Is the same kind of cycling behavior possible if hh < dh < 0? It may be the case that this is possible if what is primarily driving the cycles is that low-ranking individuals can receive many dd payoffs by interaction with other lower-ranked individuals.”

Response: A key feature of our model is Roth-Erev reinforcement learning as updating mechanism for strategies and network connections. Since we use Roth-Erev reinforcement learning dynamics for updating strategy choice and network connections, we need to keep payoffs positive. Subtracting weights in a Roth-Erev updating setting can lead to bottoming out (zero weights across options) and other problems (negative weights would break the probabilistic choice feature of the dynamic). The need to use positive update values is a general feature of Roth-Erev reinforcement learning. Negative payoffs would require using a different learning rule and different updating dynamics. In short, it would require the development of an entirely different model. That said, the referee is right to recognize the role of total accumulated payoff in driving the rank cycling pattern (more on this below). We have added the below sentence in the results section to make this dependence clear (p. 10).

> “Since the payoffs are ≥ 0, individuals never strictly prefer to opt out of any interaction. This contributes to the rank cycling. Individuals are able to accumulate positive payoffs as hubs playing *dove* as host to many *hawk* visitors, thereby enabling their rise in the rankings. In effect, having more interactions tends to increase an individual's social ranking.”

Even though Roth-Erev reinforcement learning requires positive payoffs, we were able to explore rank updating dynamics with a rule that mimics negative *hh* and *dh* payoffs by subtracting a constant *c* per interaction for assessing rank changes only (the reinforcement of strategy and network weights proceeded as usual with the Roth-Erev updating). When *c=dh* this effectively treats a dh payoff as zero and hh as negative. When *c>dh*, both *dh* and *hh* are negative. When we adopt this rule for rank updating we do not see the cycling behavior because, as the reviewer predicted, agents cannot accumulate payoffs from playing dove as host to enable a rise in the ranks. Instead, we see noisy rank changes across the length of simulation (see figures below). This noise occurs because few individuals are receiving positive per-interaction payoffs (i.e., *hd* or *dd*) so rank changes for many agents become a random walk. These individuals are unable to learn their effective ranks and so play more noisily towards highly ranked individuals which introduces additional fluctuations in rank. However, the correlated convention (playing hawk away and dove at home) still emerges in these cases with

one small difference. Some (top-ranked ranked agents) agents are very noisy in their strategy choice as hosts, often oscillating between dove and hawk host plays. Visitor strategy choice quickly converges on hawk for all players. We have included below a time series for a couple different agents in a population of size 20 to illustrate both the noise in rank change and the stable emergence of the correlated convention. Given the noise in these results, and the rather artificial way we have to mimic negative payoffs to work with the Roth-Erev learning rule we adopt in our model, it is not clear what value would be gained by including these results in the supplemental information. However, it is possible to include these results, if the reviewer and editor deem it appropriate.

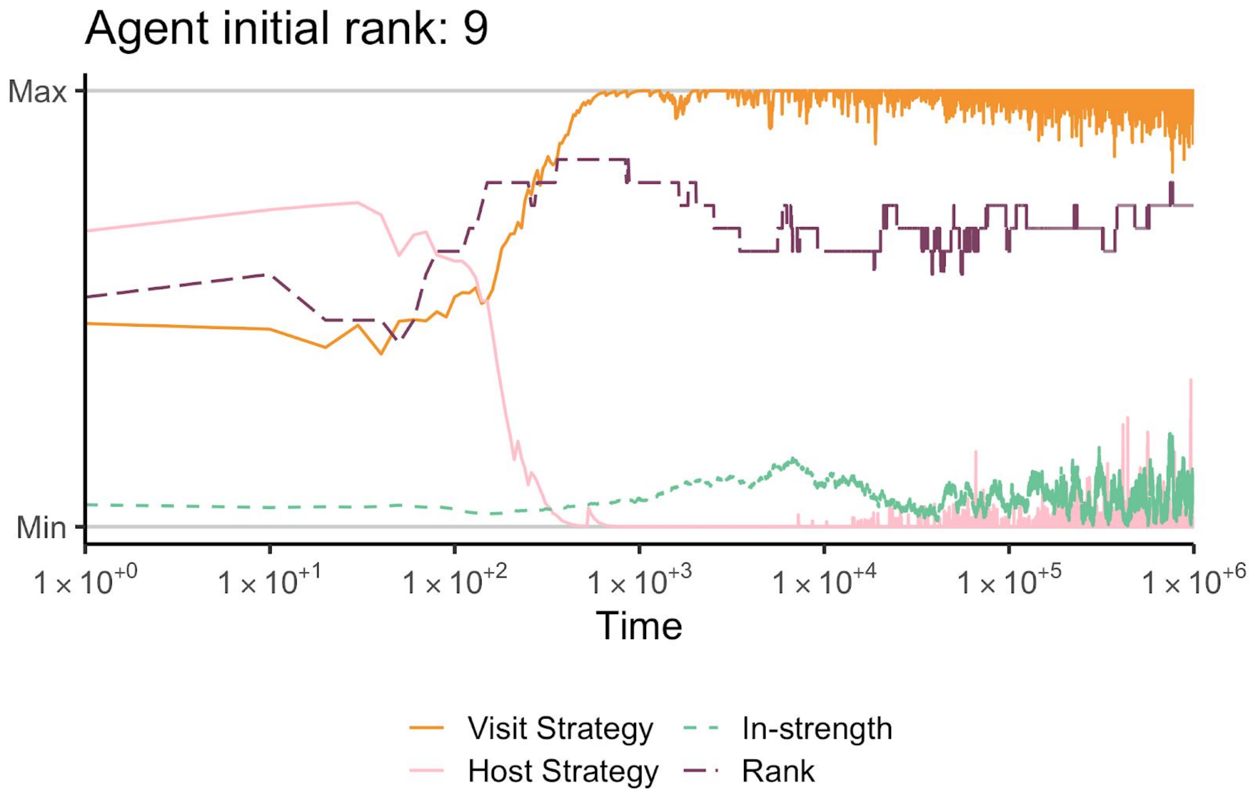

Figure 1. Do cycles persist when agents cannot accumulate resources from positive payoffs? Example dynamics from a single simulation shows noisy rank changes. Despite adopting a dove-at-home strategy the agent does not rise to the top rank without the ability to accumulate payoffs. Figure shows results for agent initially seeded at Rank 9 with: c = dh; f = 0.4; dh = 0.4; dd=0.6. Results look essentially similar for *c = dh + 0.1*, for different values of f, and for agents of different initial rank.

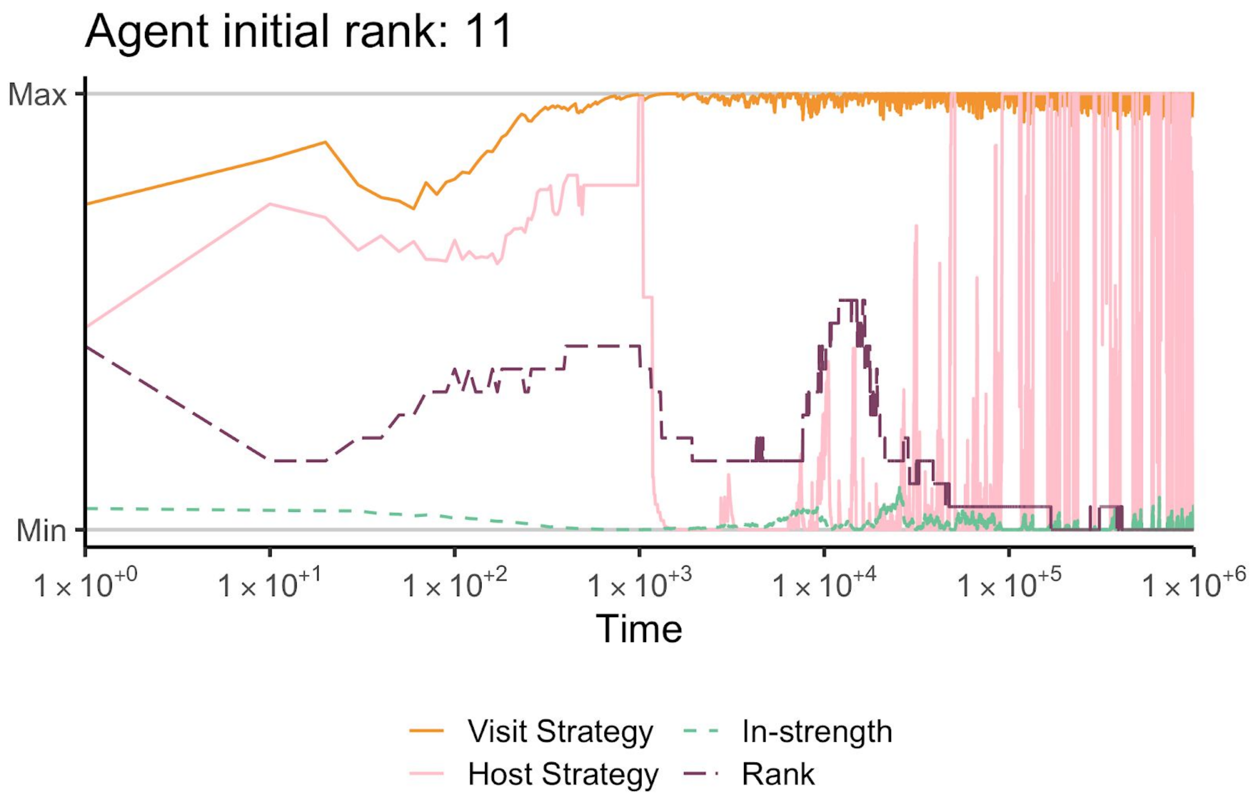

Figure 2. Example of a different agent from the same simulation. Agents with top ranks often are very noisy in their strategy choice as hosts, often oscillating between dove and hawk host plays (c = dh; f = 0.4; dh = 0.4; dd=0.6).

R#1-2. On a similar note, how do the dynamics of this model depend on the relative value of the payoffs 2(dd) received from the interaction of two doves and dh + hd received from the interaction of a hawk and a dove? Does this change the social welfare of the long-time outcome, or alter the ability to maintain the coordinating-convention rather than splitting into pure hawk and pure dove behaviors.

Response: As you can see from our new analyses above, the emergence of the correlated convention does not critically depend on the exact payoffs used in our simulation: even when agents cannot accumulate any resources, the correlated convention still emerges. Long-term social welfare remains high, as the majority of interactions follow the convention (i.e., close to the (theoretical) baseline of the correlated convention). However, the noisy rank changes lead to more aggressive hawk-hawk "collisions" which introduce friction in the overall population. As a result, long-term social welfare is lower in cases with noisy rank changes.

Regarding the case of 2dd>dh+hd, it is true that higher social welfare could be earned by more (dove, dove) play. However, as long as hd>dd, *dove-dove* is not an equilibrium and consequently reinforcement learners will never converge on this behavior with one another. Changing the payoffs to satisfy this inequality would not significantly change the outcomes of the model. While it may affect early play by introducing more transient dd-dd plays, the

correlated convention should still invade. As a result, among the possible equilibria of the game, dh+hd remains the optimal social welfare point and hence is a fitting basis of comparison.

R#1-3. In addition, I have several comments on the presentation of the payoff matrix and for the analysis of the static game.

a. I believe the payoff matrix (in table 1) should be presented much earlier in the paper, so that references to the payoff parameters in the main text are more

$$\begin{array}{cc} & \begin{array}{cc} \text{hawk}_j & \text{dove}_j \end{array} \\ \begin{array}{c} \text{hawk}_i \\ \text{dove}_j \end{array} & \begin{pmatrix} f, hh & hd, dh \\ dh, hd & dd, dd \end{pmatrix}. \end{array} \quad (1)$$

clearly understood without having to first read the appendix.

Response: This is a great idea. We now introduce the payoff matrix earlier in the main text.

b. I don't think the use of the symbol ∨ appropriately conveys the asymmetry of payoffs for players with different ranks. If, without loss of generality, we consider the interaction with players whose ranks satisfy i < j, one can present the payoffs to an i (row) and j (column) player through the following asymmetric payoff matrix

Formulations of similar asymmetric games in which either both players face a Hawk- Dove game or in which one player faces a Prisoners' Dilemma and the other a Hawk-Dove game are studied in the paper of McAvoy and Hauert [1] or by Hall et al [2].

Response: We have followed both your suggestions. First, we present the payoffs in the asymmetric way you suggest -- and as mentioned above, we do so earlier in the paper. Second, thank you for suggesting the McAvoy & Hauert [1] and Hall et al. [2] references. We have added citations to those papers in the text.

c. If, in addition, the assumption that hh = 0 is delayed for the static game analysis, one can derive more general conditions for the relative ranking of individuals required for an individual to have dominance solvability as a pure hawk player. In particular, the condition of Equation 9 becomes

$$P(\text{hawk}_j) < \frac{hd - dd}{hh - dd - (Rf + (1-r)hh - dh)}, \quad (2)$$

and then individuals who outrank the fraction R of the population should play pure hawk when

$$R > R^* = \frac{dh - hh}{f - hh}. \quad (3)$$

Therefore the threshold dominance probability $R* < 1$ when $dh < f$, agreement with the special case in which $hh = 0$. In particular, the fact that this is true even when payoff values are potentially negative provides some more motivation for understanding whether the cycling behavior is still possible if $dh < 0$ and whether the dynamics seen under partner choice can be achieved through high total payoff or high average payoff across interactions.

Response: Given our response to the negative payoff point above, introducing the assumption that hh = 0 allows for easier expressions. While we agree with your insight that without the hh = 0 assumption, we could keep the expression more general in the way you suggest, we prefer to keep the expression simpler. The general conditions are implied. Our analytic model is designed to help interpret the Roth-Erev reinforcement learning results, so the more general expression is not helpful and we prefer to retain the simpler expression.

R#1-4. The mechanism governing the partner choice and the subsequent maintenance of the coordinating convention with static rankings or cyclic behavior with dynamical ranking differs from the mechanisms often studied on the evolution of cooperation in adaptive networks. Mechanisms like active linking [3] or assortative interactions [4] tend to help form clusters of cooperators who attain higher average payoffs than defectors who end up interacting with other defectors. It could for the authors to provide more description of how the behaviors in this model differ from how previous work has studied evolution of cooperation on adaptive networks for both Prisoners' Dilemma games and games of conflict.

Response: Thank you for encouraging us to expand the discussion of our contribution. We added the following sentences, including citations to the two papers you mention, at the end of our discussion section.

The majority of models of network formation do not involve strategic choice but just a stochastic process of forming ties [22,23], active linking [58] or assortative interactions [59]. Models that do allow for strategic choice assume that the basis on which choices are founded remain static thus resulting in stable (possibly unique) equilibrium networks [60,61,62,63]. None of these models captures realistic dynamics such that more powerful individuals adopt more aggressive strategies and others may choose to avoid interaction with them. As a result, none of the existing models can explain cycles in the data we observe. Our work is part of a family of models being developed now that can explain richer patterns of network formation [64], in particular the realization that the maintenance of network ties requires effort and is dependent on positive utility derived from those interactions.

R#1-5. It may also be relevant to discuss the extent to which certain behaviors require the host-guest paradigm of interactions. For the static analysis, the two main results about of high-ranking individuals facing a Prisoners' Dilemma problem for sufficiently large f and that low-ranking individuals then see all-dove as the best-response against a population with many all-hawk individuals can be realized through a single type of interaction. In addition, the cycling behavior could possibly still arise under a similar partner choice scheme where each player chooses one opponent and plays one strategy against their opponent of choice as well as individuals that choose them for interactions. For the model with partner choice and static rankings, how would allowing only a single kind of interaction affect the long-term levels of cooperation (as the correlated-convention is no longer possible). If any of the behaviors do or do not require the host-guest framework, it would be helpful to discuss why this is the case.

Response: Without the ability to distinguish between host and visitor roles (and play different strategies as host or visitor), it is not possible for agents to learn correlated conventions (since there is no external cue to anchor the correlation of their behaviors). Instead there would be convergence to a mixture of hawks and doves: the doves receiving more visitors and thereby accumulating more payoffs, gaining rank and then becoming hawks, losing visitors and beginning cycles. However, we expect there to be significant noise and mixtures of strategies without high levels of cooperation. Killingback and Doebeli (Proc. Biol. Sci. 263[1996]: 1135-1144) have a similar result. They showed that hawk-dove games played on spatial structures lead to increased (dove-dove) cooperation but there is still significant noise and hawk-hawk collisions. In a way, this highlights that despite the noise in ranks, and cycling of behavior, you get this constant high level of cooperation due to the emergence of the convention.

R#1-6. "Correspondingly, the discussion on page 20 for the breakdown of the correlated-convention can probably be simplified. Equations 14 and 15 seem potentially redundant given the information discussed before Equation 16."

Response: The referee makes a good point about the redundancy of these equations. We have cut them.

Minor Comments

R#1-7. For the purpose of illustrating the competition over shared resources that typify games of conflict, I think it may be useful to present some parametrizations of the asymmetric payoff matrix (either when you introduce the general payoff matrix, or in the methods section or an appendix). One paramterization of potential interest for this paper would be the payoff matrix considered by Hall et al in models of repeated snowdrift games in which individuals have unequal ability to win a fight, yielding asymmetric payoff

from the hawk-hawk interaction [2].

Response: Done. We have changed how we present the payoff matrix. Instead of presenting the matrix as part of Fig. 1, we now present the payoff matrix earlier in the paper and we do so as an asymmetric payoff matrix as you suggest -- which is similar to the format used in the Hall et al. paper you reference.

R#1-8. In the methods section, it would be useful to understand how the weights for network interactions are updated based on the payoffs from previous interactions. It would probably suffice to include analogues of Equations 2 and 3 that describe how strategies depend on payoff.

Response: Done. We have expanded the Methods section to provide details and equations that describe network updating. In doing so, we have rearranged some parts of the Method section slightly to avoid redundancies and make the exposition easier to follow.

R#1-9. In several places, the term fitness function is used to describe the expected payoff for playing strategies like hawk or para. The term fitness probably should not be used here, because strategy updates depend on reinforcement learning rather than social imitation or birth-death events, and the term expected payoff is actually what is more directly being computed in the static analysis anyway.

Response: You are right, we should not use the term “fitness” in our paper. We have made careful revisions and removed the term from the paper (in particular several instances in the static model section).

R#1-10. Another potentially relevant reference for games of conflict with partner choice is a paper by Tsvetkova and Buskens which considers models and experiments on chicken games in which individuals can choose their network neighbors [5].

Response: This is indeed an interesting study with a form of asymmetry in which individuals can choose their partner, and can play different actions against different partners. We have added this paper to our literature review table. Thank you for suggesting it.

R#1-11. There is a fair bit of repetition in the paper. I particularly notice several iterations of the explanation of the cycling behavior (which potentially provides important intuition) and multiple observations about how the threshold behavior $f > dh$ does not depend on population size. For the latter comment, it is useful to know that the dynamics are not strongly dependent on population, but is also probably not necessary to overemphasize this overation after considering that ranking individuals see hawk as a dominant strategy precisely when $f > dh$, setting into motion the need for others to respond to the all-hawk actions of high-ranking individuals.

Response: We have carefully revised the entire manuscript to reduce repetition and improve its readability overall.

R#1-12. For Figure 14, the axis labels are missing. The plot is understandable from the description above, but it may still help to revise the figure.

Response: Done. We have updated the plot with proper labels.

Response to Reviewer 2

R#2-0. [...] Thereby, I am happy to recommend the acceptance of this paper in its current form.

Response: Thank you for taking the time to be a reviewer on our paper. We were excited to hear you liked our paper and recommended accepting it. We addressed your minor comments and thank you for suggesting these improvements.

Minor Comments

R#2-1. The first two sentences in Introduction are exactly the same as in Abstract. It's better to use a different expression.

Response: Done. We have revised the opening paragraph to avoid using the same sentences as in the abstract.

R#2-2. The resolution of Fig.1 b is poor in the current version. Also, it's better to add what the node size represents in the caption of Fig.1 b.

Response: Done - we increased the resolution of the embedded network graphs. In conjunction with another reviewer's comment we have generally revised Figure 1 and have now moved the summary figure to the supplement.

R#2-3. I find the non-monotonous phenomenon in Fig 3 a and b where the highest proportion of pure hawks emerges at intermediate values of f is interesting and I notice that the authors have tried to provide an intuitive explanation (Line 220-221, 225-227). However, I feel that it can be better explained as a combined effect of both avoiding conflicts with ranking individuals and avoiding less benefits from hawk-dove interactions with lower-ranked individuals. The latter is caused by partner choice: lower-ranked individuals would reduce visits to a higher-ranked Hawk host.

Response: We are glad the reviewer finds this interesting, as we did, too, when the results emerged. We appreciate the opportunity to discuss the point in more detail. What we tend to see is this: at low f values (<0.5) it doesn't really pay for ranking individuals to learn hawk so the correlated convention endures across the population. As f increases then we start to see top ranking individuals become pure hawks and specifically target out-ranked individuals. Then, as f approaches 1, we see the ranking individual stops to distinguish who to visit. All other individuals then get visits from the top-ranking individual and are pushed back towards dove as host.

We have attempted to add some more explanation around this point in the result section on page 7:

The reason for this is that, as f approaches 1, the alpha (top-ranking) individual stops

distinguishing among all other individuals. This individual simply learns to always play hawk and all possible hosts are seen as equivalent. At the same time, additional early hawk behavior from ranking individuals may lead lower-ranked individuals to avoid visits from others. Consequently, ranking individuals (with the exception of the alpha) have higher proportions of hawk visitors compared to lower f and therefore have a greater incentive to play dove when hosting.

R#2-4. some minor mistakes:

Line 18: is dangerous work -- is a dangerous work
Line 76: in that that -- in which
Line 226: at least at least -- at least
Line 615: when does does – when does

Response: Done - we fixed those mistakes and also carefully proofread the entire manuscript to make sure it is without errors.

Response to Reviewer 3

Overall Response: Thank you for taking the time to be a reviewer on our paper. We have made several improvements to the paper overall. In particular we have improved the organization (e.g., moved some details from the main text to the Methods section), improved the clarity of writing, and avoided unnecessary repetitions. We addressed your other comments below.

> **R#3-1.** Time scales. There are several dynamics: the rank and the network, and the strategy. Previous studies show [66,69] that the time scale of the strategy dynamics and the underlying network would greatly alter the cooperation level. In this work in the method, the authors only adopt one time scale (line 513) for rank dynamics. How Robust is the result with the time scale of different dynamics? A discussion along this line would benefit the audience.

Response: Thank you for pointing to the importance of time scales on different dynamics. Our original manuscript already included detailed investigation of time scale dynamics in the supplement in the Section *Learning speed comparison* (p. 24). However, these were only mentioned briefly in the main text and, based on your comment, we have revised and expanded the discussion of time scales in the main text (Section *Power Asymmetries and Partner Choice* and Section *Dynamic Power Asymmetries*) and in the Supplementary Information (p. 24). We find all our results are robust to different time scales. In the case of our model with static ranks, even very slow network updating (1/10000th of strategy updating) is sufficient to restore the correlated convention (Fig. S9). In the case of dynamic ranks, network learning speed affects cycle length, but cycles continue to emerge even with slow network learning (Fig. 10).

> **R#3-2.** Fig 1 is not illustrative enough, I am not clear from the figure how network evolves, and how individuals adjust their behaviors.

Response: We have revised the conceptual part of Figure 1 to provide a more comprehensive overview over the step of gameplay and model updating. This is now shown as the new, stand-alone Figure 1. We removed the summary part (previously Figure 1b) from the paper entirely since it, confusingly, focused on baseline results such as random interaction without bullies, rather than the core findings of the paper.

> **R#3-3.** “Convention” is not defined in a clear way. Typically convention refers to the believing system (e.g. A thinks that B is good if B helps C), here, it seems to refer to a resulting population composition. It should be clearly defined.

The referee is correct that we do not explicitly define convention. This was an oversight on our part. We have revised the introduction to make clear that we are adopting a common definition from game theory. We have added the below sentence in the 4th

paragraph in the introduction:

> *Since these correlated equilibria identify patterns of behavior that are ``customary, expected, and self-enforcing'' [13], they are identified as paradigm examples of conventions in game theory [14, 15].*

R#3-4. The interaction between [power] asymmetry and network paves the way (intuitively) to an oscillatory dynamics. (line 385-391), how on earth that this oscillatory dynamics lead to an resilient convention? An intuitive understanding would benefit.

Response: Intuitively, the host-guest convention minimizes the impact of the presence of bullies (since, with that norm, everyone accommodates aggressive visitors anyway). The presence of bullies is all that matters and not *who specifically* the bully is. While a small number of high-ranking individuals adopt aggressive strategies, the majority of the population settles on the host-guest norm. With this norm, the lower-ranked individuals are equally impacted by visits from bullies as from similarly ranked individuals. With dynamic ranks, some individuals slowly cycle out of the cooperative cluster and adopt aggressive strategies. The cooperative cluster itself remains intact and continues to isolate whoever is the bully at the time. So what leads to a resilient convention despite the oscillatory dynamics is the fact that bullies only make up a small proportion of the overall population and partner choice (the dynamic network) allows the population to continuously ostracize whoever is the bully at any given moment. We've added additional explanation to the discussion at the point you suggested.
